# Breathing adapted radiotherapy: a 4D gating software for lung cancer

**DOI:** 10.1186/1748-717X-6-78

**Published:** 2011-06-24

**Authors:** Nicolas Peguret, Jacqueline Vock, Vincent Vinh-Hung, Pascal Fenoglietto, David Azria, Habib Zaidi, Michael Wissmeyer, Osman Ratib, Raymond Miralbell

**Affiliations:** 1 Department of Radiation Oncology, University Hospital, Geneva, Switzerland; 2 Department of Nuclear Medicine, University Hospital, Geneva, Switzerland; 3 Department of Radiation Oncology, CRLC Val d'Aurelle, Montpellier, France

**Keywords:** Lung cancer, radiotherapy, 4D-CT, gating

## Abstract

**Purpose:**

Physiological respiratory motion of tumors growing in the lung can be corrected with respiratory gating when treated with radiotherapy (RT). The optimal respiratory phase for beam-on may be assessed with a respiratory phase optimizer (RPO), a 4D image processing software developed with this purpose.

**Methods and Materials:**

Fourteen patients with lung cancer were included in the study. Every patient underwent a 4D-CT providing ten datasets of ten phases of the respiratory cycle (0-100% of the cycle). We defined two morphological parameters for comparison of 4D-CT images in different respiratory phases: tumor-volume to lung-volume ratio and tumor-to-spinal cord distance. The RPO automatized the calculations (200 per patient) of these parameters for each phase of the respiratory cycle allowing to determine the optimal interval for RT.

**Results:**

Lower lobe lung tumors not attached to the diaphragm presented with the largest motion with breathing. Maximum inspiration was considered the optimal phase for treatment in 4 patients (28.6%). In 7 patients (50%), however, the RPO showed a most favorable volumetric and spatial configuration in phases other than maximum inspiration. In 2 cases (14.4%) the RPO showed no benefit from gating. This tool was not conclusive in only one case.

**Conclusions:**

The RPO software presented in this study can help to determine the optimal respiratory phase for gated RT based on a few simple morphological parameters. Easy to apply in daily routine, it may be a useful tool for selecting patients who might benefit from breathing adapted RT.

## Introduction

Lung cancer is the first cause of cancer death in the world with an overall 5 year survival rate inferior to 15%. It has been shown that local control after radiotherapy (RT) is dose-dependent with a better overall-survival for patients with the disease locally controlled [1-3]. Nevertheless, physiological respiratory motion of primary lung tumors may challenge the chances of obtaining an optimal local control rate after RT.

There are presently several approaches under investigation aiming to correct for tumor motion potentially leading to a better conformality of RT: tumor tracking, synchronizing the beam-on/beam-off time with respiratory motion (gating), or using 4D-CT to determine the average tumor motion during a respiratory cycle in order to define an internal target volume [4-7]. A 4D-CT acquires sets of images in different respiratory phases and can be employed for respiratory gated radiotherapy [8]. Systematic errors can thus be reduced and reliable target margins can be defined, in order to avoid the risk of underdosing due to tumor motion [9]. Respiratory gating has been shown to reduce the size of the planning treatment volume (PTV) defined by 4D-CT and is expected to improve the therapeutic ratio by raising the dose to the tumor and decreasing the dose to the surrounding normal tissues [10,11].

Although there are techniques compensating for respiratory motion during RT and delivering RT during one specific moment of the respiratory cycle, the optimal moment for delivering RT remains unknown and controversial. Irradiation during deep inspiratory breath hold (DIBH) is considered by some to have dosimetric advantages in terms of lung sparing through the inspiratory expansion of the healthy lung tissue [12,13]. However, DIBH may not be feasible in patients with compromised pulmonary function. On the other hand, end-expiration is considered to be more reliable by others because it is longer and more reproducible than end-inspiration [14].

In this report we present a respiratory phase optimizer (RPO) for breathing adapted RT (BART) in order to determine the optimal irradiation phase based on a few simple morphological parameters.

## Methods and Materials

Fourteen patients with a primary or recurrent lung cancer were retrospectively studied. 4D-CTs were acquired during 4 to 6 respiratory cycles for every patient in the study. Patient data sets were provided by the Geneva University Hospital (6 cases), the CRLC Val d'Aurelle (6 cases), and by the General Electric Corporation (2 cases).

**Figure 1:**
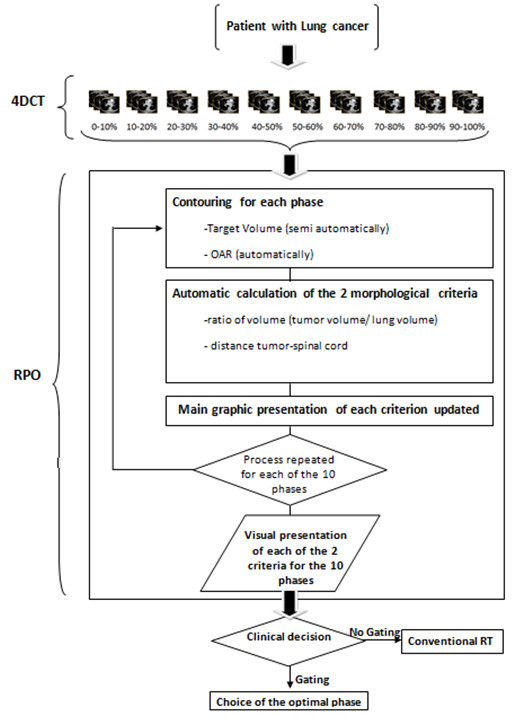
**Flowchart of the process**.

Ten 4D-CT axial images corresponding to ten time bins (phases) of the respiratory cycle (i.e., in 10% increments) were reconstructed, using a maximum intensity projection (MIP) system (Figure 1). The MIP (maximum intensity projection) is a visualization method for 3 D imaging data. It was first described by Wallis *et al*. originally called "maximum activity projection", for nuclear medicine use [15]. It is now widely employed in radiology and in particular for 4D-CT [16]. During the 4D image acquisition, the scan extracts information continuously during a time interval equivalent to a breathing cycle. After that, and using an external physiological signal, the ADW (advantage workstation) system can reconstruct retrospectively 10 CT sets, each of them representing an acquisition on the same breathing phase. Therefore, our cam gets 10 CT-scans equivalent to 10 breathhold positions. For the same slice coordinates, 10 different values for the same voxel in the DICOM reference are obtained. A MIP can be created by building a new image, looking for the maximum value of the 10 different scans in the corresponding voxel. In Geneva, the MIP system was implemented by a commercial software provided on the Biograph TP 64 scanner (Syngo software, Siemens Medical Solutions, Erlangen, Germany). A time reference for the 4D image datasets was obtained with the Real-Time Position Management system (RPM, Varian Medical Systems Inc., Palo Alto, CA) for 8 patients and the Anzai system (Anzai AZ-733V system, Anzai Medical Co, Ltd., Tokyo, Japan) for 6 patients.

**Figure 2:**
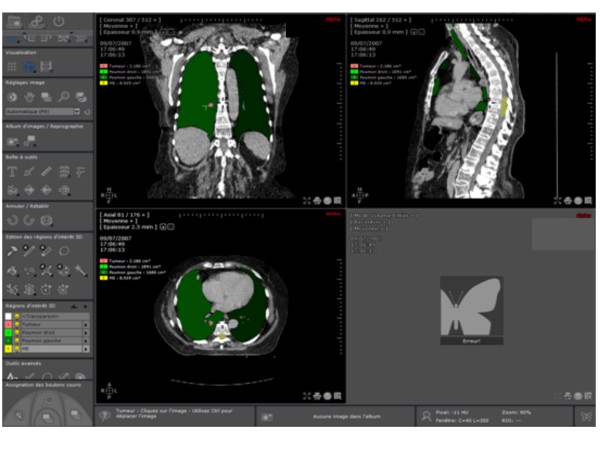
**Visualization of all the "ROI" necessary to calculate the criteria of comparison**.

As shown in Figure 1, two parameters were defined to compare the images of each phase: a) the target to lung volume ratio (T/L ratio_vol_), ideally as small as possible and b) the tumor to spinal cord distance (T-C_dist_), sought to be as large as possible. A low T/L ratio_vol _may obviously result in optimal target coverage with a simultaneous reduced lung irradiation. DIBH has shown the potential for a reduced lung V20 (i.e., percent of lung volume receiving 20 Gy) [12]. Choosing the phase where T-C_dist _is the largest is based on the fact that dose constraints to the spinal cord have the highest priority in ongoing trials [17]. An image processing software ("Myrian^®^", developed by the Intrasense Company, Montpellier, France) was used for delineation and volume determination of the tumor and OARs (Figure 2). The external limits of the target and of the OARs were defined on images derived directly from a DICOM CD to work with usable cross sections. All the contouring was done by the same author (NP). The segmentation of the gross tumor volume (GTV) and of the OARs (lungs and spinal cord) is done by "Myrian^®^" semi-automatically and automatically, respectively. This process results in the definition of four regions of interest (ROI): the GTV, the right lung, the left lung and, the spinal cord.

Because "Myrian^®^" is not able in the current version to calculate T/L ratio_vol _and T-C_dist_, all these data are then transferred to the RPO, where their calculations and graphical presentation are automatized for each respiratory phase. In this process, "Myrian^®^" is unable to perform an automatic propagation of TV and OAR delineated from one phase to the other nine. So, this sequence is repeated for each of the ten phases of the respiratory cycle and with the CT data acquired in maximal inspiration (the reference). Once the data are collected, the RPO is able to display a bar graph for both comparison parameters: the T/L ratio_vol _and the T-C_dist_. A graph displays, in addition, the absolute ipsilateral lung volume measured at each respiratory phase. A synoptic summary of the two graphs is presented to the user who may then proceed to the assessment of the optimal respiratory phase.

For the present study the percentual difference between the optimal respiratory phase and maximal inspiration (the reference) was assessed. If both were coincident, we computed, in addition, the percentual difference between the optimal respiratory phase and the least optimal one. We considered that there was no gain if the difference was ≤ 20%.

## Results

Patients and tumor characteristics are presented in Table 1. The output of the RPO for each individual case is presented in additional file 1: RPO_appendice.doc. Table 2 and Table 3 present, respectively, (T/L ratio_vol_) and (T-C_dist_) for the 14 patients according to the ten sequential respiratory phases chosen in our study.

**Table 1:** Patients and tumor characteristics

Patient	Age	Sex	Site	Stage	Histology	**Mean GTV volume (cm**^**3**^**)**
**1**	unknown	M	Right middle lobe	T2N0M0	unknown	90

**2**	unknown	F	Left lower lobe	TXN2M0	unknown	403

**3**	46	M	Right paratracheal	TXN3M0	SCC	27

**4**	51	M	Right upper lobe	T2N2M0	NSCLC	86

**5**	75	F	Right lower lobe	T1N0M0	AC	2

**6**	75	F	Right lower lobe	T1N0M0	AC	2

**7**	71	M	Right upper lobe	T1N0M0	unknown	7

**8**	64	F	Right upper lobe	T1N0M0	AC	10

**9**	62	M	Left lower lobe	T3N0M0	unknown	2

**10**	81	F	Left lower lobe	T2N1M0	SCC	68

**11**	65	M	Left upper lobe	Stage IV (M1)	SCC	37

**12**	81	M	Right middle lobe	T2N1M0	SCC	66

**13**	70	M	Right upper lobe	T1N0M0	AC	6

**14**	63	F	Right lower lobe	Extensive. disease	SCLC	10

Table 1: SCC = squamous cell carcinoma, NSCLC = non-small cell lung cancer, AC = adenocarcinoma, SCLC = small cell lung cancer.

**Table 2:** Tumor to lung volume parameter (T/L ratio_vol _= 100* Tumor volume/Ipsilateral lung volume)

Patient	Respiratory phase										Phase opt = ref	% gain opt/ref	% gain opt/worst
				
	0-9%	10-19%	20-29%	30-39%	40-49%	50-59%	60-69%	70-79%	80-89%	90-99%			
**1**	1.90	2.00	1.60	2.00	1.90	1.90	2.20	2.00	1.90	1.90	no	20	27
**2**	33.5	35.8	44.7	56.6	65.5	71.7	71.1	59.0	45.8	37.6	yes	0	53
**3**	1.20	1.10	1.20	1.00	1.20	1.30	1.20	1.30	1.20	1.20	no	17	23
**4**	5.10	6.00	5.90	6.00	6.70	6.40	6.40	6.6	6.00	5.50	yes	0	24
**5**	0.09	0.06	0.08	0.13	0.14	0.15	0.12	0.08	0.07	0.13	no	33	60
**6**	0.12	0.04	0.11	0.16	0.14	0.19	0.12	0.10	0.09	0.17	yes	67	79
**7**	0.25	0.23	0.22	0.22	0.22	0.23	0.24	0.24	0.24	0.24	no	12	12
**8**	0.30	0.30	0.36	0.41	0.44	0.43	0.42	0.44	0.42	0.31	yes	0	32
**9**	0.11	0.11	0.09	0.09	0.09	0.11	0.12	0.12	0.12	0.12	no	0	25
**10**	5.50	5.60	5.80	5.90	5.90	6.70	6.30	5.90	5.70	6.10	yes	0	18
**11**	2.75	2.15	1.76	1.86	1.53	1.90	2.15	1.95	2.18	2.64	no	29	44
**12**	5.50	5.70	5.20	5.60	4.70	3.80	4.50	4.70	4.80	5.30	no	31	33
**13**	0.26	0.26	0.26	0.27	0.25	0.32	0.32	0.32	0.31	0.26	no	4	22
**14**	0.40	0.42	0.47	0.48	0.40	0.44	0.52	0.44	0.40	0.40	yes	0	23

Phase where maximal inspiration was observed is indicated by underlining

Ref = maximal inspiration, opt = optimal phase found by RPO, worst = worst phase found by RPO

**Table 3:** Tumor to spinal cord distance parameter (T-C_dist_) in mm.

Patient	Respiratory phase										Phase opt = ref	% gain opt/ref	% gain opt/worst
				
	0-9%	10-19%	20-29%	30-39%	40-49%	50-59%	60-69%	70-79%	80-89%	90-99%			
**1**	165	164	163	167	170	167	168	166	165	164	no	4	4
**2**	66	67	66	68	69	68	67	69	66	67	no	5	5
**3**	63	61	57	60	60	58	61	60	58	58	yes	0	11
**4**	75	91	95	91	65	70	70	72	79	74	no	27	46
**5**	43	41	42	41	41	40	40	38	37	40	yes	0	16
**6**	40	43	41	41	44	43	39	41	40	42	no	10	13
**7**	115	115	114	115	115	114	116	113	115	115	no	1	3
**8**	126	129	129	128	127	128	128	126	126	127	no	2	2
**9**	72	72	72	71	72	70	71	72	71	71	no	0	3
**10**	47	50	50	48	47	47	51	51	49	49	no	9	9
**11**	74	70	72	70	70	71	73	72	70	71	no	6	6
**12**	61	60	58	61	63	61	64	63	64	68	no	11	17
**13**	83	84	84	84	84	84	84	84	85	84	no	2	2
**14**	92	90	89	88	89	90	90	90	88	88	yes	0	5

Phase where maximal inspiration was observed is indicated by underlining

Ref = maximal inspiration, opt = optimal phase found by RPO, worst = worst phase found by RPO

As also shown in Table 2, maximal inspiration occurred mostly at the beginning of the 4D-CT recording: phase 0-9% in 9 patients and 10-19% in 4 patients. Only in patient #9 maximal inspiration occurred during the phase 60-69% of the respiratory cycle. Concerning the optimal T/L ratio_vol_, the optimal respiratory phase coincided with maximal inspiration in only 6 cases. The mean difference between the optimal respiratory phase and maximal inspiration (the reference) was 15% (SD ± 19) ranging from 0 (optimal phase coinciding with maximal inspiration) to 67%. Compared to the worst phase of the respiratory cycle, the mean difference between the optimal phase and the less optimal one was 34% (SD ± 18) ranging from 12 to 79%.

Regarding the second parameter, the T-C_dist_, the optimal phase coincided with maximal inspiration in only 3 cases (Table 3). The mean difference between the optimal respiratory phase and maximal inspiration was 5.5% (SD ± 7.0) ranging from 0 to 27%. Compared to the worst phase of the respiratory cycle, the mean difference between the optimal phase and the less optimal one was 10% (SD ± 11) ranging from 2 to 46%.

**Figure 3:**
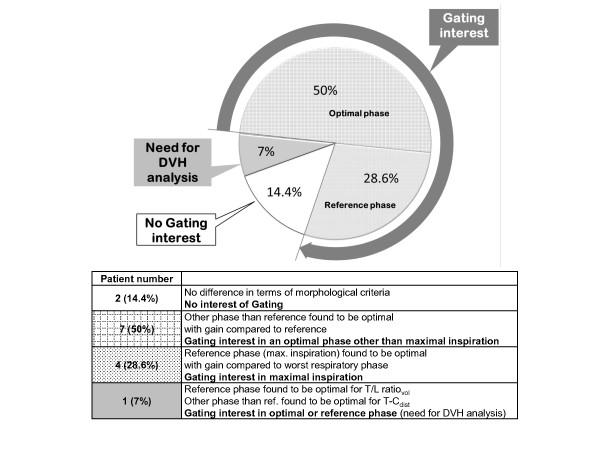
**Overview of morphological results and their interpretation**.

With a cut-off of 20% only 2 cases showed no benefit in either of both parameters (patients #7 and #10). In 11 patients, however, a substantial gain was observed for the T/L ratio_vol_, the optimal phase coinciding with maximal inspiration in 4 (28.6%) and differing from maximal inspiration in 7 (50%). In only one patient (7%) (Patient #4) maximal inspiration was optimal for the T/L ratio_vol_, but was suboptimal for the T-C_dist _Figure 3 displays the corresponding overall summary.

## Discussion

Physiological respiratory motion is a major challenge for lung cancer RT. The range of motion can reach an average up to 12 ± 6 mm for tumors in the lower lung lobes [18]. Giraud *et al*., observed large diaphragm displacements in the cranio-caudal direction during free breathing with an average range of 34 mm and a maximum of 67 mm between inspiration and expiration. Reduced motion, however, has been reported for tumors in the lung apices with an average of 8 mm displacement in the cranio-caudal direction between inspiration and expiration [19]. A patient's breathing pattern varies from day to day (inter-fraction motion) and can vary during an individual RT fraction (intra-fraction motion) [20]. As a consequence of respiratory motion, planning target volume (PTV) margins in the order of 1.5-2 cm are commonly used for RT without breathing control. These margins increase, obviously, the irradiated lung volume and consequently the risk of pulmonary radiation toxicity [21]. The most consistent and predictive parameters for radiation induced lung toxicity are the V20 and the mean lung dose (MLD) [22,23]. It is widely accepted that keeping V20 <30-37% and MLD <20Gy may yield a relatively low risk of pneumonitis (<20%).

Our findings are consistent with Giraud *et al*., in his analysis of intrathoracic organ motion during breathing [19]. Indeed, tumors growing in the lower lung lobes and not attached to the diaphragm (i.e., patients #2, #5, #6, #9, #10, and #14) presented with large variation of T/L ratio_vol _or T-C_dist_., translating in a potential benefit from respiratory gating techniques. Giraud *et al*., observed also that the smallest displacements were in the apices and near the tracheal carina. This is in agreement with our observation that centrally located tumors may benefit less from gating based on the present algorithm, especially when fibrous attachments to the mediastinum restrict their mobility (e.g., patient #3). Five tumors growing in the superior lung regions (i.e., patients #4, #7, #8, #11, and #13) presented less, though not negligible, changes in the chosen comparative parameters. For patients with tumors growing in the posterior mediastinum, close to the spinal cord, the RPO helped to find the optimal respiratory phase other than maximal inspiration (i.e., patient #4). Maximum inspiration, the reference, was optimal in only 28.6% of cases (Figure 3). In 50%, however, other phases of the respiratory cycle were found to be optimal as identified by RPO.

Although, gating techniques are reasonably time consuming, and they may not be needed for every patient. A threshold of tumor motion or tumor volume needs to be defined above which gating can be recommended. Starkschall *et al*., found that patients with small tumors (GTV <100 cm3) benefitted the most from gating [24]. Therefore, the RPO software may also help to identify patients with minimal tumor motion influence for whom a gating-free treatment can be recommended.

Easy to apply in daily routine, fast in getting the optimization result, and no special hardware needed are the main practical advantages of the RPO worth to be highlighted. It is important, however, to plan on a 4D-CT to be able to acquire synchronized image sets. Data analysis represents about 2000 calculations (volumes, densities, surfaces, inertia axes, density histograms, ratio of volumes, and distances) for every patient.

Variability in target volume delineation is a major source of error in 4D-CT treatment planning. Because all the contours were defined by the same author, inter-observer variability was unavailable in our study in response to the need of technique novelties claimed in some recent literature in the 4D-CT era [25]. In a new version of the Myrian software, a contour propagation tool has been integrated which is expected to reduce intra-observer variability, but the accuracy of this tool needs to be investigated in a dedicated study before implementation in clinical routine.

An evident limitation of our study is the reduced number of patients studied so far and the restricted morphological parameters of the comparison not including dose-volume parameters in the analysis. Nevertheless, it seems reasonable to assume a dosimetric gain when treating patients in the optimal respiratory phase selected by the RPO.

Further development of the presented software is planned in order to adapt it for tumor locations in the upper abdomen as treatment reproducibility may also be conditioned by respiratory motion. In addition, the density histograms obtained with "Myrian^®^" may also be used to assess the treatment response after treatment.

## Conclusion

The RPO software presented in this study can help to determine the optimal respiratory phase for gated RT based on a few simple morphological parameters. Easy to apply in daily routine, it may be a useful tool for selecting patients who might benefit from BART.

## Competing interests

The authors declare that they have no competing interests.

## Authors' contributions

NP conceived the RPO software, provided and cared for study patients, performed all target volume and OAR delineation, contributed to data acquisition and drafted the manuscript. JV contributed to the study design, provided and cared for study patients, contributed to data acquisition and revised the manuscript critically. VVH contributed to the presentation of our results and revised the manuscript critically. PF contributed to the study design (in particular the choice of the morphological criteria), and provided Montpellier patient data. DA contributed to the study design (in particular the choice of the morphological criteria) and provided cooperation with CLRC Val d'Aurelle. HZ conceived and introduced the use of low dose 4DCT in Geneva and contributed to data acquisition. MW provided collaboration with the Nuclear Medicine department in Geneva and provided and cared for study patients in the Nuclear Medicine department. OR provided collaboration with the Nuclear Medicine department in Geneva and assumed the overall responsibility from the Nuclear Medicine department. RM permitted to NP to develop this study in the Radiation Oncology department in Geneva, revised the manuscript critically and assumed the overall responsibility for the study. All authors read and approved the final manuscript.
